# Acrolein and Asthma Attack Prevalence in a Representative Sample of the United States Adult Population 2000 – 2009

**DOI:** 10.1371/journal.pone.0096926

**Published:** 2014-05-09

**Authors:** B. Rey deCastro

**Affiliations:** Centers for Disease Control and Prevention, National Center for Environmental Health, Division of Laboratory Sciences, Atlanta, Georgia, United States; Tsinghua University, China

## Abstract

**Background:**

Acrolein is an air toxic and highly potent respiratory irritant. There is little epidemiology available, but US EPA estimates that outdoor acrolein is responsible for about 75 percent of non-cancer respiratory health effects attributable to air toxics in the United States, based on the Agency's 2005 NATA (National-Scale Air Toxics Assessment) and acrolein's comparatively potent inhalation reference concentration of 0.02 µg/m^3^.

**Objectives:**

Assess the association between estimated outdoor acrolein exposure and asthma attack reported by a representative cross-sectional sample of the adult United States population.

**Methods:**

NATA 2005 chronic outdoor acrolein exposure estimates at the census tract were linked with residences oif adults (≥18 years old) in the NHIS (National Health Interview Survey) 2000 – 2009 (n = 271,348 subjects). A sample-weighted logistic regression model characterized the association between the prevalence of reporting at least one asthma attack in the 12 months prior to survey interview and quintiles of exposure to outdoor acrolein, controlling for potential confounders.

**Results:**

In the highest quintile of outdoor acrolein exposure (0.05 – 0.46 µg/m^3^), there was a marginally significant increase in the asthma attack pOR (prevalence-odds ratio [95% CI]  = 1.08 [0.98∶1.19]) relative to the lowest quintile. The highest quintile was also associated with a marginally significant increase in prevalence-odds (1.13 [0.98∶1.29]) in a model limited to never smokers (n = 153,820).

**Conclusions:**

Chronic exposure to outdoor acrolein of 0.05 – 0.46 µg/m^3^ appears to increase the prevalence-odds of having at least one asthma attack in the previous year by 8 percent in a representative cross-sectional sample of the adult United States population.

## Introduction

Acrolein is among the 189 pollutants designated under the 1990 Clean Air Act [1] to be a hazardous air pollutant (or air toxic) known to or suspected of causing cancer or other serious health problems. A simple aldehyde, acrolein (CAS 107-0-28; IUPAC prop-2-enal) is a potent irritant of the respiratory tract, including the nose and throat [2]–[5]. Acrolein can also irritate the eyes and skin [6], [7]. Acrolein was deployed as a chemical warfare agent (tear gas) at least once during World War I [8]–[11].

Acrolein is used mainly as a precursor in the manufacture of acrylic acid and as a biocide. Acrolein is ubiquitous in the environment, and since it is formed through the combustion of petroleum (especially diesel fuel), acrolein is a concern for mobile sources, aircraft, and industrial boilers [12]. Another major component of mobile emissions — 1,3-butadiene — oxidizes in the atmosphere to acrolein, further augmenting acrolein's presence in the environment. US EPA (United States Environmental Protection Agency) estimates that approximately three-quarters of ambient acrolein exposure originates from mobile sources [13]. Acrolein exists in the atmosphere in the vapor phase, and is subject to photochemical degradation by hydroxyl radicals, ozone, and nitrate radicals. Acrolein's half-life in the atmosphere based on observed gas-phase photolysis indicates a half-life of 10.9 days [14], but calculated half-life estimates range from 3.4 hours to 28 days depending on the type of photolytic reaction [15]–[20].

Acrolein is also a major component of the vapor phase of tobacco smoke, as well as smoke from cooking, forest fires, and biomass combustion. Small amounts of acrolein have also been detected in some foods, such as fried foods, cooking oils, and roasted coffee [6], [20]–[22]. Acrolein is also formed endogenously as part of physiological oxidative stress response and polyamine metabolism [23].

Although direct evidence of acrolein's carcinogenicity in humans or experimental animals is considered inadequate [24], acrolein is known to induce DNA damage [25] and to form DNA adducts relevant to lung cancer and inhibition of tumor suppression [26], [27]. Acrolein has also been shown to interact with a prominent carcinogenic constituent of tobacco smoke — benzo[*a*]pyrene — to inhibit p53 tumor suppressor activity, which suggests a role for acrolein in lung cancer initiation [28].

Attention has focused recently on the potential role of endogenous acrolein — produced as part of oxidative stress response — in a variety of neurologic disorders, such as Alzheimer's disease, Parkinson's disease, and amyotrophic lateral sclerosis [29]–[32]. Endogenous acrolein has also been observed in connection to spinal cord injury [33], as well as myelin damage [34]. The possibly adverse neurological effects of endogenous acrolein have prompted concern about environmental exposure to acrolein, particularly through air pollutant emissions and tobacco smoke.

Acrolein's role as a respiratory toxicant is well established [6], [7]. Because of its high reactivity with human tissue, inhalation of acrolein has been hypothesized to induce or exacerbate acute lung injury and chronic obstructive pulmonary disease [21]. A risk assessment for human lung function extrapolated from rat data suggested that ambient concentrations of acrolein in the United States may be associated with reduced respiratory function [35]. In a comprehensive review considering the exposure prevalence and toxic potency of hazardous air pollutants, acrolein was recommended for further research into its role in the initiation and exacerbation of asthma [36]. Other risk assessments of air toxics have singled out acrolein as a prominent noncancer respiratory health risk in the United States [37], [38] and in Pittsburgh, PA [39].

Asthma is a chronic inflammatory disorder of the small respiratory airways. Acute episodes, or asthma attacks, in susceptible individuals are associated with airflow obstruction characterized by wheezing, breathlessness, chest tightness, and coughing. These episodes result from a combination of airway inflammation and elevated bronchial hyperresponsiveness to a variety of triggers. Important environmental triggers include ETS (environmental tobacco smoke), dust mites, cockroach allergen, outdoor air pollution, wood smoke, pets, and mold [40], [41]. As a potent respiratory irritant, acrolein may have a prominent role as an environmental trigger of asthma attacks. Asthma attacks are usually reversible, either spontaneously or with medication, and while there is no cure for asthma, symptoms can be managed through medication and the avoidance of environmental triggers [42]–[44].

There are standard US EPA methods for measuring ambient acrolein (Method TO-11a, based on a canister sampler, and TO-15, a cartridge method), with temporal resolution of hours to days [45], [46]. Negative bias has been reported with Method TO-11a [47]. A real-time quantum cascade laser infrared absorption device is also commercially available [48]. Because acrolein is highly reactive, ambient measurement remains notoriously difficult, which increases uncertainty about the consistency and reliability of acrolein monitoring data [49], [50]. Improving measurement of ambient acrolein is the subject of ongoing research [51], [52].

With the difficulties in measuring ambient acrolein, it is not surprising that there is scant epidemiologic research into the health effects of acrolein in general populations. In striking contrast to this lack of information, US EPA estimates that acrolein is responsible for about 75 percent of non-cancer respiratory health effects attributable to air toxics in the United States, based on the Agency's NATA (National-Scale Air Toxics Assessment) for 2005 [13]. Acrolein's environmental ubiquity notwithstanding, its prominence in risk assessments like NATA and others [35]–[39] is driven primarily by its distinctively high potency for respiratory health effects compared to other air toxics, with a chronic inhalation RfC (reference concentration) of 0.02 µg/m^3^ [53].

The aim of this study was to provide empirical evidence of the association between acrolein exposure and respiratory irritation, as denoted by the prevalence of self-reported asthma attacks among adults in the general population. This was achieved by geographically linking NATA 2005 acrolein exposure concentration estimates at the census tract level with residences of participants in NHIS (National Health Interview Survey) 2000 – 2009 [54]. NHIS data have been geographically linked to US EPA ambient air monitoring data to examine the associated prevalence of childhood asthma [55] and respiratory allergies [56], [57], but this is the first time that NHIS data have been linked to NATA exposure estimates. Geographically linked NATA data has also been used to study air toxics exposure and asthma in a children's birth cohort [58], cancer [59], and autism spectrum disorder [60], [61].

## Materials and Methods

### National-Scale Air Toxics Assessment 2005

NATA 2005 estimates chronic inhalation exposure risks for 179 air toxics, and is a tool for prioritizing geographic areas, pollutants, and emission sources for further evaluation of public health impact [13]. US EPA developed NATA to augment data from the Agency's nationwide air toxics monitoring network by taking a model-based approach to quantifying the potential health risks from air toxics. NATA provides risk estimates for each of the approximately 66,000 census tracts comprising the United States. Census tracts are delineated by the US Census Bureau, and each typically contains about 4,000 residents and ranges from 1,500 to 8,000 residents. Tracts also vary in geographic size, with urban tracts usually smaller than two square miles and rural tracts being much larger [62].

NATA encompasses outdoor emissions of air toxics from stationary point sources, non-point sources, and mobile sources (on-road and off-road), as well as estimates of background concentration and secondary formation of acrolein from the decay of 1,3-butadiene. In 2011, US EPA released NATA 2005 [13] based on 2005 National Emissions Inventory data [63], which were the most complete and up-to-date inventory data at the time of the analysis. NATA 2005 combines data from the National Emissions Inventory and US EPA's air monitoring system as inputs to air dispersion and photochemical models (American Meteorological Society/EPA Regulatory Model [AERMOD], Assessment System for Population Exposure Nationwide Model [ASPEN], and Community Multiscale Air Quality Model [CMAQ]) to estimate outdoor air toxics concentrations. The air dispersion models account for the physical topography and population density in each census tract. The outdoor concentration estimates are then used as the basis for estimating chronic air toxics exposure concentrations from a screening-level inhalation exposure model (Hazardous Air Pollutant Exposure Model, Version 5: HAPEM5). These exposure concentrations account for differences in exposure resulting from demographics, human activities, commuting patterns, climate data, and indoor-to-outdoor variability. The NATA process concludes with estimation of cancer and non-cancer public health risks [64], but for the analyses reported here only the acrolein inhalation exposure concentrations estimated at the census tract level were used. And, unlike NATA health risk estimates, NATA exposure concentration estimates do not utilize unit health risk factors like the RfC.

### National Health Interview Survey 2000 – 2009

NHIS is conducted by NCHS (National Center for Health Statistics) of the CDC (Centers for Disease Control and Prevention) and is a principal source of information on the health conditions, behaviors, and access to health services of the civilian, non-institutionalized population of the United States. Information is collected through a comprehensive interview of a nationally representative cross-section of households [65]. NHIS is designed to produce national estimates of disease prevalence, and provides reliable data for estimating asthma prevalence across the United States, over time, and across subgroups [66]. Sampling is conducted annually following a multistage area probability design that permits the representative sampling of households and noninstitutional group quarters (e.g., college dormitories). This study evaluated the cross-sectional prevalence of having at least one self-reported asthma attack in the 12 months prior to each subject's interview, which represented affirmative answers to both NHIS questions “Have you ever been told by a doctor or other health professional that you had asthma?” and “During the past 12 months, have you had an episode of asthma or an asthma attack?” This definition identifies subjects with symptomatic asthma and is the standard CDC definition for evaluating asthma attack prevalence [55].

Additional NHIS data on potential confounders were evaluated in the logistic regression models: sex, age, race/ethnicity, poverty status, education, health insurance coverage, access to health care, whether the subject's residence was located in an urban or rural area, as well as NHIS interview year and calendar quarter. Acrolein is a component of tobacco smoke, so smoking status (never smoked, former smoker, and current smoker) was evaluated as a potential confounder and separate models were fit for never smokers and never & former smokers. Information for potential confounders was reported by the participant or an adult in the participant's household, except for poverty and insurance status, urban residence, survey year, and calendar quarter. Poverty status of each subject's family was determined according to a calculated PIR (poverty income ratio): the ratio of family income to the US Census poverty threshold adjusted for family size. Families with a PIR <1.00 were categorized as poor. The uninsured were subjects who did not have health insurance at the time of the interview under private health insurance, Medicare, Medicaid, State Children's Health Insurance Program, a state-sponsored health plan, other government programs, or military health plan. Insurance status for the subject may have been reported by an adult in the subject's family. This definition of uninsured matches that used in Health United States [67]. Access to health care was assessed through the NHIS question “Is there a place that you usually go to when you are sick or need advice about your health?” Relevant places include hospital emergency rooms and outpatient departments. Residence in an urban or rural area followed US Census Bureau classification and is available from restricted-use NHIS data files.

### NATA-NHIS Linked Data and Statistical Models

One adult 18 years of age or older was randomly selected from each participating NHIS family to provide detailed information about their health, including symptomatic asthma and smoking status. This study compiled ten years of data (2000 – 2009) regarding these sample adults, numbering 287,530 subjects [54]. Of these, 9,524 subjects were missing census tract information, and another 6,658 subjects were missing data on the outcome or predictors. This left 271,348 subjects (94.4 percent) who were included in the logistic regression models reported here. The Census 2000 tract identifiers for NHIS subjects's residences are available from restricted-use NHIS data files, which enabled assignment of subjects to their respective census tracts. Using Census 2000 tract delineations, NATA acrolein exposure data at the census tract were geographically linked with health outcome data for individual NHIS subjects.

Since NHIS is a complex survey in which subjects are obtained through a multistage sampling design involving stratification, clustering, and oversampling of specific population subgroups, the complex design must be accounted for in order to ensure properly estimated variances of regression coefficients obtained from statistical models of NHIS data [66], [68]. Robust estimation of these variances may be accomplished through a generalized estimating equations approach incorporating Taylor series linearization and applying sampling weights to each survey subject. This approach was used as it was implemented by the CROSSTAB, DESCRIPT, and RLOGIST subroutines of SUDAAN version 10.0.1 [69] called from the SAS version 9.3 statistical software application [70]. To parameterize the variance estimation procedure, variables indicating the NHIS sampling stratum and primary sampling unit were used. Pursuant to NCHS guidance [71], [72], sampling strata were renumbered to distinguish strata used in different NHIS sample design periods: 2000 – 2005 and 2006 – 2009. In addition, the final annual sample weights from the NHIS 2000 – 2009 sample adult files (WTFA_SA) for subjects 18 years old and older were adjusted for incomplete data (see below). To yield annualized totals, sample weights were divided by ten, which accounts for combining data across ten NHIS sampling years [71], [72].

Sample-weighted logistic regression models were fit to the linked NATA-NHIS data where the dichotomous outcome was whether an NHIS subject answered affirmatively that they had both ever been diagnosed with asthma and had at least one asthma attack in the previous 12 months. The reference outcome group comprised NHIS subjects who: a) answered affirmatively that they had ever been diagnosed with asthma, but answered negatively that they had had at least one asthma attack in the previous 12 months; and b) answered negatively that they had ever been diagnosed with asthma. The predictor of interest was the quintile of NATA 2005 chronic inhalation exposure concentration to acrolein for the census tract where the NHIS subject resided. Additional predictors representing potential confounders (described above) were evaluated in the regression models.

Acrolein is a prominent component of tobacco smoke, so it is important to assess acrolein's influence on asthma attack prevalence among subjects who do not smoke. This was evaluated in two sample-weighted logistic regression models for NATA-NHIS 2000 – 2009 subjects limited to those reporting: a) that they never smoked (“never smokers”; n = 153,820); and b) that they either never smoked or were former smokers (“never & former smokers”; n = 212,537). No subjects in these strata were current smokers, but the second, larger stratum enabled us to evaluate the potential influence of discontinued smoking. The predictors in both stratified models were identical to the unstratified, except that the stratified models did not control for smoking status. Statistical significance was set at α≤0.05, and marginal significance was set at 0.05<α≤0.15.

Among all NHIS 2000 – 2009 sample adults, 24.0 percent were missing information on PIR, which derives from the historic difficulty of obtaining self-reported income data. To address this problem, NCHS provides multiply imputed data on income and PIR. Specifically, for each subject, NCHS provides public-use PIR data that have been imputed five times [54], which were included in the statistical analyses reported here. Statistical analysis of multiply imputed data yields multiple results that can be combined to obtain unified variance estimates that reflect the additional variation due to the multiple imputation procedure [73]. Earlier evaluation of the performance of multiply imputed NHIS income data indicated that it corrects biases that would otherwise occur without multiple imputation and that statistical efficiency is improved [74]. Consistent with NCHS guidance [75], this approach was adopted through the use of the MI_VAR option in the CROSSTAB and RLOGIST subroutines of SAS-callable SUDAAN version 10.0.1. All results reported here and the sample size described above are based on multiply imputed PIR data.

Regression models require complete information on all dependent and predictor data for each subject, which were available for 271,348 of 287,530 subjects (94.4 percent) in the NATA-NHIS linked data. Since subjects with incomplete data were not part of the statistical models reported here, model estimates would have been biased to the degree there was non-randomness in the pattern of missing data. To ameliorate this potential bias, the original NHIS survey sample weights (WTFA_SA) were adjusted prior to statistical analysis using the WTADJUST subroutine of SAS-callable SUDAAN version 10.0.1, which treats those subjects with complete information as a random subsample of all NHIS subjects. In this approach, the probability of an NHIS subject being selected into this subsample was assumed to be a logistic function of covariates. The parameters of this logistic selection model were estimated implicitly by WTADJUST in adjusting the sample weights. This approach is pursuant to current NCHS guidance [76], and because all models reported here used sample weights adjusted in this manner, results may be considered statistically representative of the United States civilian, noninstitutionalized adults comprising the NHIS study population [77].

### Ethics Statement

The NCHS Research ERB (Ethics Review Board) protected the rights and welfare of participants in the NHIS. In accordance with Federal regulations, the NCHS ERB reviewed and approved NHIS protocols and any changes made to them. This process ensured the ethical treatment of NHIS participants. Documented signed informed consent was obtained from each participant.

## Results

**Figure 1:**
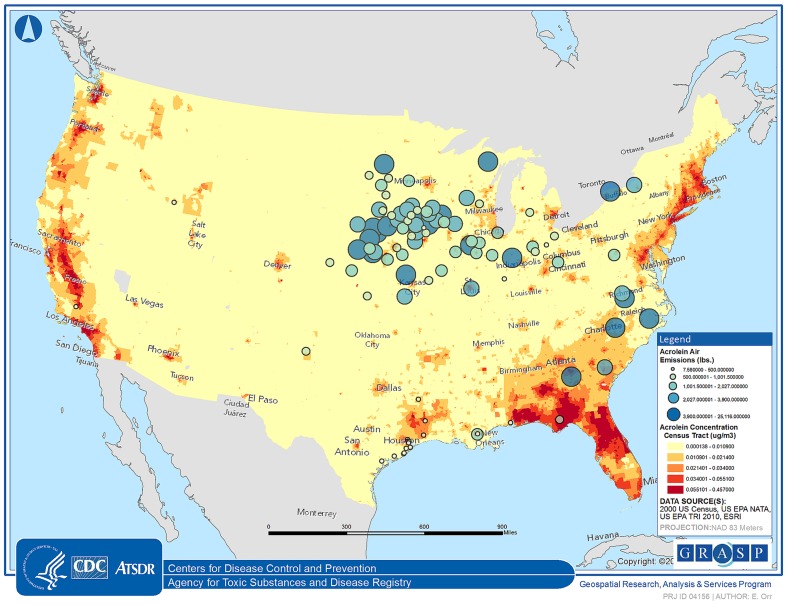
Map of the contiguous United States indicating the NATA 2005 acrolein exposure concentration quintile [μg/m3] for each census tract (shaded areas) overlaid with locations of 102 facilities reporting on-site air releases of acrolein [lbs] to the 2010 Toxic Release Inventory (bubble size corresponds to quintile of acrolein released).

Figure 1 displays the NATA 2005 acrolein exposure concentration quintile for each census tract in the contiguous United States overlaid with locations of 102 facilities that reported to the 2010 Toxic Release Inventory [78] (accessed through TOXMAP [79]) that they released acrolein to the air on-site categorized by the quintile of the amount released. This map demonstrates how acrolein exposure is more widespread than suggested by the locations of emitting facilities, which is consistent with the prominence of mobile sources for acrolein exposure.

Table 1 displays the sample-weighted proportion of subjects across acrolein exposure quintiles and across demographic characteristics among the 271,348 adult subjects comprising the NATA-NHIS data used in the logistic regression models. The national sample-weighted prevalence of 12-month asthma attacks was 3.8 (standard error: 0.1) percent among adults during 2000 – 2009. Because assignment of subjects to acrolein exposure quintiles did not account for sample weighting, the sample-weighted distribution of subjects among acrolein quintiles reported in Table 1 do not appear even. Table 2 summarizes the distribution of outdoor acrolein exposure concentrations: the sample-weighted median acrolein chronic inhalation exposure concentration was 2.5E-2 (IQR: 3.2E-2) µg/m^3^, with a range from 1.4E-4 to 4.6E-1 µg/m^3^.

**Table 1:** Characteristics of subjects comprising NATA-NHIS 2000 – 2009 data: 271,348 of 287,530 subjects (94.4 percent) with 3.8 (0.1) percent (standard error) prevalence of at least one asthma attack in previous 12 months (sample-weighted).

Predictor	Sample-Weighted Percent (SE)
Acrolein Exposure Quintile	
1st: 1.4E-4 –1.1E-2 µg/m3	21.7 (0.5)
2nd: 1.1E-2 – 2.1E-2	21.9 (0.5)
3rd: 2.1E-2 – 3.4E-2	20.7 (0.5)
4th: 3.4E-2 – 5.5E-2	18.8 (0.4)
5th: 5.5E-2 – 4.6E-1	16.8 (0.4)
Sex	
Female	51.9 (0.1)
Male	48.1 (0.1)
Age	
18 – 24 years-old	13.0 (0.2)
25 – 34	18.0 (0.1)
35 – 44	20.0 (0.1)
45 – 54	19.2 (0.1)
55 – 64	13.6 (0.1)
65 – 74	8.7 (0.1)
75 – 84	5.8 (0.1)
≥85	1.8 (0.04)
Race/Ethnicity	
Hispanic	12.4 (0.2)
Non-Hispanic Black/African American only	11.3 (0.2)
Non-Hispanic Other	5.5 (0.1)
Non-Hispanic White only	70.8 (0.3)
Smoking Status	
Current Smoker	21.7 (0.1)
Former Smoker	21.9 (0.1)
Never Smoked	56.5 (0.2)
Education	
High School Graduate or Higher	83.2 (0.2)
Less than High School Graduate	16.8 (0.2)
Poverty Income Ratio (Imputed)	
1.00 or Higher	89.2 (0.1)
<1.00 (Poor)	10.8 (0.1)
Place to Go When Sick	
No place	14.7 (0.1)
One or more places	85.3 (0.1)
Residence	
Rural	25.0 (0.5)
Urban	75.0 (0.5)
NHIS Interview Year	
2000	9.4 (0.1)
2001	9.5 (0.1)
2002	9.6 (0.1)
2003	9.9 (0.1)
2004	10.0 (0.1)
2005	10.1 (0.1)
2006	10.2 (0.1)
2007	10.4 (0.1)
2008	10.5 (0.1)
2009	10.6 (0.1)

**Table 2:** Sample-weighted statistics of outdoor acrolein exposure concentrations for NATA-NHIS 2000 – 2009 data.

Statistic	Quantity [μg/m^3^]
Mean (SE)	3.4E-2 (3.6E-4)
Geometric Mean (geometric standard error)	2.3E-2 (2.3E-4)
Median (interquartile range)	2.5E-2 (3.2E-2)
5th Percentile	4.9E-3
10th	6.6E-3
25th	1.2E-2
75th	4.4E-2
90th	6.9E-2
95th	8.7E-2

Representative of the United States civilian, noninstitutionalized adult (> = 18 years) population.

Based on the sample-weighted logistic regression model for NATA-NHIS 2000 – 2009 subjects reported in Table 3 (comprising the same data described in Table 1), there was a marginally significant (p-value = 0.10) increase in the 12-month asthma attack pOR (prevalence-odds ratio) of 1.08 [95% CI (95 percent confidence interval): 0.98∶1.19] at the highest exposure quintile (5.5E-2 to 4.6E-1 µg/m^3^) relative to the lowest quintile (1.4E-4 to 1.1E-2 µg/m^3^), controlling for sex, age, race/ethnicity, poverty status, education, smoking status, access to health care, whether the subject's residence was located in an urban or rural area, and NHIS interview year. The reference group for this model was 35 – 44 year old, non-Hispanic white, non-smoking males, with one or more places to go for medical care or advice, and educational attainment of a high school diploma or more, who were interviewed in 2005, and were living in an urban household with a poverty index ≥1.00. Health insurance coverage and NHIS interview quarter were initially included in the model, but since they were not statistically significant they were dropped. For the second through fourth acrolein exposure quintiles (1.1E-2 to 5.5E-2 µg/m^3^), pORs ranged from 1.01 to 1.02 and were statistically indistinguishable from asthma attack prevalence in the lowest quintile of acrolein exposure.

**Table 3:** Adjusted prevalence-odds ratios for having at least one asthma attack in the previous 12 months among a representative cross-sectional sample of adult (≥18 years-old) United States population.

	All Subjects		Never Smokers		Never & Former Smokers	
	n = 271,348		n = 153,820		n = 212,537	
Acrolein Exposure Quintile [μg/m3]	pOR (95% CI)	p-Value	pOR (95% CI)	p-Value	pOR (95% CI)	p-Value
1st: 1.4E-4 – ≤1.1E-2	Reference		Reference		Reference	
2nd: >1.1E-2 – ≤2.1E-2	1.02 (0.94, 1.12)	0.61	1.07 (0.93, 1.22)	0.35	1.03 (0.92, 1.15)	0.60
3rd: >2.1E-2 – ≤3.4E-2	1.02 (0.93, 1.12)	0.69	1.11 (0.97, 1.27)	0.11	1.04 (0.94, 1.16)	0.46
4th: >3.4E-2 – ≤5.5E-2	1.01 (0.92, 1.11)	0.83	0.98 (0.86, 1.12)	0.77	0.97 (0.87, 1.08)	0.60
5th: >5.5E-2 – ≤4.6E-1	1.08 (0.98, 1.19)	0.10	1.13 (0.98, 1.29)	0.08	1.09 (0.98, 1.22)	0.13

Adjusted for sex, age, race/ethnicity, poverty income ratio (imputed five times), education, access to health care, whether a subject's residence was located in an urban or rural area, and NHIS interview year. The unstratified model for all subjects also controlled for smoking status, while the stratified models for never smokers and never & former smokers did not.

Among adult never smokers and never & former smokers (Table 3), the prevalence-odds of having at least one asthma attack in the previous 12 months was 13 percent [1.13, 95% CI: 0.98∶1.29] and 9 percent [1.09, 95% CI: 0.98∶1.22] higher at the highest acrolein exposure quintile compared to the lowest quintile. These elevated prevalence-odds, however, had marginally significant p-values of 0.08 and 0.13. Additionally, there was a marginally significant 11 percent increase in asthma attack prevalence-odds in the third acrolein exposure quintile compared to the lowest (p-value = 0.11), but this pattern was confined to never smokers.

## Discussion

In a representative cross-sectional sample of the adult civilian, non-institutionalized population of the United States for 2000 – 2009, chronic residential exposure to outdoor acrolein (estimated for 2005) at the highest quintile of 0.05 – 0.46 µg/m^3^ was found to increase the prevalence-odds of having at least one asthma attack in the previous 12 months relative to the lowest quintile by about 8 percent at a marginally significant level, as estimated in a sample-weighted logistic regression model and controlling for potential confounders. Since the NHIS data were pooled over 2000 – 2009, statistical estimates are interpretable as being averaged over the time interval of the pooled data [71], [72].

These marginally significant results among adults are worth comparing to a recent study of a nationally representative cohort of United States children followed since infancy (n = 6,950) [58]. Adopting a somewhat different approach to using the NATA estimates, this study geographically linked by zip code air toxics respiratory health risk estimates from NATA 2002 to cohort subjects in order to evaluate associated changes in asthma prevalence. This study found no evidence of significantly increased prevalence of asthma associated with total air toxics respiratory health risk. The important differences between the designs of Stoner, Anderson and Buckley [58] and the present study notwithstanding, it is notable that although both utilized long-term averages derived from NATA, neither respiratory risk exposure comprising all air toxics [58] nor ambient concentration exposure for acrolein was strongly associated with increased asthma prevalence. This may point to the larger difficulty of associating short-term, acute health episodes with long-term average exposure rather than, say, coincident real-time exposure measurements that captures highly resolved temporal spikes in exposure more relevant to the health outcome.

The highest quintile of acrolein exposure concentration was more than double the chronic inhalation RfC of 0.02 µg/m^3^ [53] and encompassed California OEHHA's (Office of Environmental Health Hazard Assessment) chronic REL (reference exposure level) of 0.35 µg/m^3^ [80]. The acrolein exposure concentration quintile encompassing the RfC — the second quintile of 0.01 – 0.02 µg/m^3^ — was not associated with significantly increased asthma attack prevalence-odds. The importance of these comparisons should be viewed in light of the uncertainty factor for acrolein's RfC, which is 1000 [53], and REL, which is 200 [80].

Elevated rates of adverse respiratory health effects, including asthma, have been widely observed among residents, especially children, living near heavily trafficked roadways [81]–[87]. Adverse respiratory health effects have been also correlated with traffic-related air pollutants like particulates, nitrogen dioxide, and sulfur dioxide [88]–[91]. Acrolein is a constituent of the complex mixture of traffic-related pollutants that are implicated in short-term, respiratory (non-cancer) health effects like asthma attacks. Since traffic density is correlated with urbanization, competing effects from other traffic-related air pollutants were accounted for by including urban residence as a model predictor, but this statistical adjustment is likely incomplete. While acknowledging these considerations, acrolein is distinguished among air toxics because of its high potency for respiratory (non-cancer) health effects. Among the 85 substances that have inhalation RfCs listed on US EPA's Integrated Risk Information System [53], only two have more toxic RfCs: chromium (VI; aerosol) and 1,6-hexamethylene diisocyanate. Twenty-seven substances have RfCs that are within two orders of magnitude less toxic than acrolein's, including 1,3-butadiene, acrylonitrile, and chromium (VI; particulate), but these three air toxics are of concern because of their cancer health risks [37], contrasted with the non-cancer health risk posed by acrolein. And while some studies have been put forward to support a proposal to relax acrolein's RfC from 0.02 to 0.62 µg/m^3^ (0.27 ppb) [92]–[94], acrolein would still be a major concern among air toxics at this proposed level.

Acrolein is a component of tobacco smoke, but both stratified non-smoker models suggest that outdoor acrolein's adverse effect on asthma attacks does not appear to be driven solely by acrolein inhaled directly from smoking tobacco.

Although it would have been desirable to also evaluate the influence of ETS, during 2000 – 2009 NHIS only asked about ETS in 2005. Restricting analysis to a single year of data would have curtailed statistical power so severely as to hinder proper evaluation of ETS as a potential predictor of asthma attack prevalence.

Median outdoor 24-hour measurements of acrolein at downtown urban sites (Pittsburgh, PA) ranged from 0.11 – 0.14 µg/m^3^ [39]. NATA focuses on outdoor sources of acrolein, and so ignores influences of indoor origin, such as cooking and building materials. Acrolein levels in indoor air, however, have been observed to be higher than outdoors. In a comprehensive quantitative literature review of indoor hazardous air pollutants in United States residences [95], the median concentration for acrolein was estimated to be 0.84 µg/m^3^, nearly double the maximum estimated outdoor exposure concentration in NATA 2005. Logue, McKone, Sherman and Singer [95] also concluded that acrolein should be considered a priority chronic and acute hazard among chemical air contaminants. Using a mist chamber technique, indoor acrolein measured in nine California homes in the morning averaged 4.0 (standard deviation: 2.2) µg/m^3^, while simultaneous outdoor measurements averaged 0.58 (0.64) µg/m^3^ [96]. In the same study, substantial diurnal fluctuation in indoor acrolein ranging up to a factor of 2.5 was correlated with cooking and ambient temperature. In a model-based exposure assessment of the United States population, nonsmokers exposed to indoor ETS by living with a smoker were estimated to be exposed to acrolein in a range from 1.6 to 3.6 µg/m^3^ [97]. Evaluated with respect to the marginally significant increase in asthma attack prevalence-odds reported here in the acrolein range of 0.05 – 0.46 µg/m^3^, the higher indoor acrolein levels described above suggest that indoor exposure may further increase asthma attack prevalence in the general population.

In order to link NATA acrolein exposure estimates with NHIS asthma data, NHIS subjects living in the same census tract were assigned the same NATA acrolein exposure concentration. This approach, however, ignores: a) intra-tract exposure variation; b) exposure subjects may have obtained in other Census tracts; c) differences in time subjects resided in a census tract. In the temporal domain, NHIS subjects were assigned the same 2005 acrolein exposure estimate across all NHIS surveys from 2000 to 2009, which ignores temporal variation in acrolein exposure. Short-term, transient emissions profiles — such as startups, shutdowns, malfunctions, and upsets — are not represented, so NATA's chronic exposure estimates for acrolein would tend to underestimate total and acute exposure. In addition, estimates from NATA 2005 do not reflect long-term trends in air toxics exposure, as would be expected as air pollution regulations for mobile and fixed sources are implemented and as older facilities are retired.

NATA comprises a rigorous and complex process in environmental health risk assessment encompassing the entire United States. US EPA has emphasized, however, that NATA is a tool primarily for prioritizing air toxics, emissions sources, and geographic areas based on health risks [64]. This prioritizing information, rather than being the final assessment of risk, instead enables regulators to target resources for additional, more refined studies. The NATA process itself is also continually refined, and although NATA 2005 incorporated improvements in data and modeling, US EPA has noted that further improvements are possible in several areas to reduce uncertainty: emissions data quality, monitoring data quality, representativeness of meteorologic data, representativeness of background air toxics concentrations, and atmospheric model formulation [64]. In addition, in a quantitative comparison of model-based NATA 2005 estimates with ambient monitoring data [49], NATA 2005 accurately predicted ambient concentrations for several air toxics, but there appears to be a tendency for NATA to underestimate concentrations for others, which may be attributable to emissions sources missing from the National Emissions Inventory, under-estimation of emission rates, bias in monitoring techniques, and difficulties in estimating background concentrations. Still, other studies indicate that NATA estimates are reasonably reliable estimates of personal exposure to ambient air toxics [98], [99]. The limitations of NATA estimates described above would contribute to exposure misclassification, adding a degree of uncertainty to the significance of the estimated association between outdoor acrolein exposure and asthma attack prevalence.

A distinct feature of NHIS is that it is conducted as a serial cross-sectional survey, hence a causative relationship between the dependent variable and the predictors cannot be concluded based on the models alone, but must be evaluated in context of dispositive epidemiologic and toxicologic evidence [100]. A particular strength of this study is the large sample size of the NATA-NHIS data, as well as its national representativeness and comprehensive detail on subject characteristics, which enabled good control of potential confounders for asthma attack prevalence in the logistic models, while at the same time examining the effect of acrolein exposure.

This is the first report suggesting an association between outdoor acrolein exposure and adverse effects on health in a general population. This was achieved through the availability of a rigorous model-based and geographically linked estimate of exposure that accounts for human activity and microenvironments. These exposure estimates also have the advantage of being at a high spatial resolution that account for variation resulting from local population density and topography. Model-based exposure estimates provide one avenue of exposure assessment, and although this report demonstrates the feasibility of conducting national epidemiologic analysis for air toxics with modeled exposure estimates, there continues to be a need for epidemiologic studies to further establish acrolein's risks in the general population. This need will be addressed as methods for measuring acrolein in ambient air continue to progress [51], [52], and as recent advances in measuring acrolein metabolites in human samples become broadly applied [101].

## Conclusion

Environmental exposure to acrolein arising from outdoor emissions in the range of 0.05 – 0.46 µg/m^3^ was associated with a marginally significant 8 percent increase in the prevalence-odds of having at least one asthma attack in the past 12 months among a representative cross-sectional sample of the 2000 – 2009 United States adult civilian, non-institutionalized population, as estimated in a sample-weighted logistic regression model controlling for potential confounders. Outdoor acrolein exposure in this range was also associated with marginally significant increases in asthma attack prevalence-odds among adults who do not directly smoke tobacco, a major source of acrolein exposure.
